# Metabolomic Analysis of Blood Plasma after Oral Administration of *N*-acetyl-d-Glucosamine in Dogs

**DOI:** 10.3390/md13085007

**Published:** 2015-08-07

**Authors:** Tomohiro Osaki, Seiji Kurozumi, Kimihiko Sato, Taro Terashi, Kazuo Azuma, Yusuke Murahata, Takeshi Tsuka, Norihiko Ito, Tomohiro Imagawa, Saburo Minami, Yoshiharu Okamoto

**Affiliations:** 1 Department of Veterinary Clinical Medicine, School of Veterinary Medicine, Tottori University, 4-101 Koyama-cho Minami, Tottori 680-8553, Japan; E-Mails: kazu-azuma@muses.tottori-u.ac.jp (K.A.); ymurahata@muses.tottori-u.ac.jp (Y.M.); tsuka@muses.tottori-u.ac.jp (T.T.); taromobile@me.com (N.I.); imagawat@muses.tottori-u.ac.jp (T.I.); saburominami@ncn-t.net (S.M.); yokamoto@muses.tottori-u.ac.jp (Y.O.); 2 Koyo Chemical Co. Ltd., 1-17 Taiyuji-cho, Kita-ku, Osaka 530-0051, Japan; E-Mails: kurozumi@koyo-chemical.co.jp (S.K.); sato@koyo-chemical.co.jp (K.S.); taro.terashi@koyo-chemical.co.jp (T.T.)

**Keywords:** amino acid, cartilage regeneration, dog, *N*-acetyl-d-glucosamine hydrochloride, TGF-beta

## Abstract

*N*-acetyl-d-glucosamine (GlcNAc) is a monosaccharide that polymerizes linearly through (1,4)-β-linkages. GlcNAc is the monomeric unit of the polymer chitin. GlcNAc is a basic component of hyaluronic acid and keratin sulfate found on the cell surface. The aim of this study was to examine amino acid metabolism after oral GlcNAc administration in dogs. Results showed that plasma levels of ectoine were significantly higher after oral administration of GlcNAc than prior to administration (*p* < 0.001). To our knowledge, there have been no reports of increased ectoine concentrations in the plasma. The mechanism by which GlcNAc administration leads to increased ectoine plasma concentration remains unclear; future studies are required to clarify this mechanism.

## 1. Introduction

*N*-acetyl-d-glucosamine (GlcNAc) is a monosaccharide that normally polymerizes linearly through (1,4)-β-linkages. GlcNAc is the monomeric unit of the polymer chitin, the second most abundant carbohydrate after cellulose. GlcNAc is commercially prepared by several companies via acid hydrolysis of crude chitin. GlcNAc is a basic component of hyaluronic acid and keratin sulfate found on the surface of cells [1].

GlcNAc is non-toxic. The half-life of GlcNAc, upon intravenous injection of 20 g, is 220 min [2]. After administration of 800 mg/os, the mean maximum concentration (Cmax) of GlcNAc in plasma was 162.7 ± 125.2 ng/mL and the time for maximum concentration (Tmax) was observed to be 1.56 ± 1.23 h [3]. It was also reported that the maximum concentration of GlcNAc in dogs reached about 4.4 μg/mL after administration of 300 mg/kg GlcNAc [4].

In recent years, GlcNAc has been found to be a valuable agent for treating a variety of diseases. We reported that GlcNAc significantly enhanced the prevention of joint damage [5] and had suppressive effects on rheumatoid arthritis in an experimental mouse model [6]. It was also reported that GlcNAc had beneficial therapeutic effects on patients with inflammatory bowel disease [7]. In fibroblasts, GlcNAc was shown to enhance proliferative capacity and increase collagen expression [8].

GlcNAc influences cell signaling through the posttranslational modification of proteins by glycosylation. O-linked attachment of GlcNAc to Ser and Thr residues modulates the expression of a variety of intracellular proteins, including transcription factors such as NFκB, c-myc, and p53 [9]. In addition, the specificity of Notch family receptors for different ligands is altered by GlcNAc attachment to fucose residues in the extracellular domain. GlcNAc also affects signal transduction by altering the degree of branching of *N*-linked glycans, which influences cell surface signaling proteins [9].

Metabolomics can be defined as an approach based on the systematic study of the complete set of metabolites present in a given biological system; it can be viewed as a mirror that reflects the physiological, evolutionary, and pathological state of a biological system. By measuring the metabolome, metabolomics enables analysis of the interaction of the genome with the environment, particularly under determined physiological conditions resulting from nutrition interventions or diet [10].

Recently, we reported a relationship between oral administration of d-glucosamine hydrochloride (GlcN·HCl) and amino acid synthesis [11]. Accelerated fumarate respiration and elevated plasma levels of lactic acid and alanine were observed after oral administration of GlcN·HCl. These results indicate that oral administration of GlcN·HCl induced anaerobic respiration and starvation in cells and further suggest that lactic acid and *O*-GlcNAc could potentially induce TGF-β production. We hypothesize that these conditions induced by oral administration of GlcN·HCl promote cartilage regeneration.

To our knowledge, this is the first report on the relationship between oral administration of GlcNAc and amino acid synthesis. The aim of this study was to examine amino acid metabolism after administration of oral GlcNAc to dogs.

## 2. Results and Discussion

### 2.1. Principal Component Analysis of Metabolic GlcNAc Profiles

**Figure 1:**
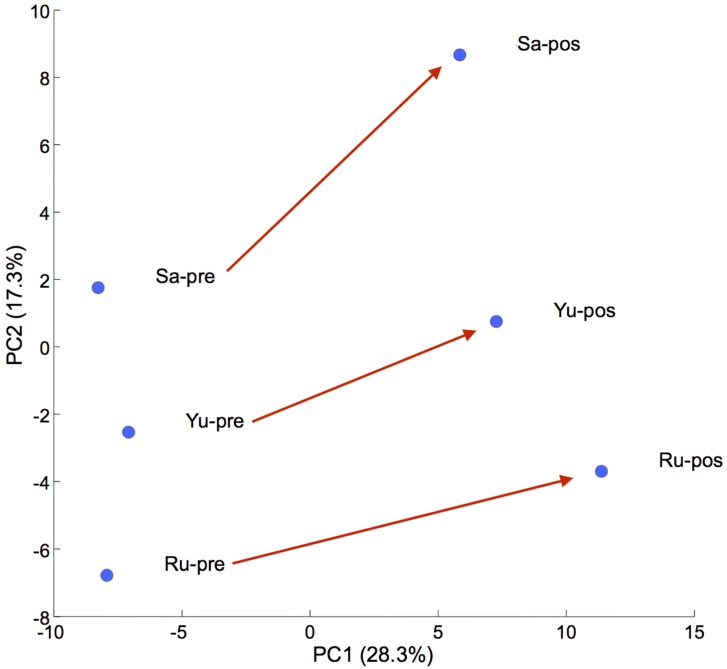
Principal component analysis (PCA). Score plots (PC1 *vs.* PC2) before (Sa-pre, Yu-pre, Ru-pre) and after (Sa-post, Yu- post, Ru- post) oral administration of GlcNAc.

Metabolome measurements were carried out by Human Metabolome Technology Inc., Tsuruoka, Japan (Commercial metabolomics analysis service). The CE-TOFMS systems in two different modes for cation and anion analyses detected 157 peaks (cation: 93, anion: 64), which were quantified. Principal component analysis (PCA) is a technique for taking high-dimensional data and, using the dependencies between variables, representing the data in a more tractable, lower-dimensional form, without losing excessive information [12]. The score plots for each dog showed similar changes when comparing scores before (Sa-pre, Yu-pre, Ru-pre) and after (Sa-post, Yu-post, Ru-post) oral administration of GlcNAc (Figure 1). PCA revealed that individual variability in GlcNAc-induced amino acid metabolism was negligible.

GlcNAc in the plasma was undetectable before and after oral administration of GlcNAc. Side effects of GlcNAc were not observed. In previous studies, when a large dose of GlcNAc (20 g) was given intravenously to human volunteers, it did not result in toxicity or alteration of blood glucose concentration [2,3]. A lack of insulin resistance was also found after oral administration of GlcNAc, even at a high dose. Therefore, GlcNAc is safe for intravenous, oral, and topical usage [1], and it was considered that GlcNAc might be widely utilized as a nutritional supplement for therapeutic dosage.

### 2.2. Changes in Plasma Metabolite Concentrations

In this study, we compared concentration of metabolites after and before oral administration of GlcNAc. All *p* values were determined by Welch’s *t* test. As illustrated in Table 1, some metabolite levels in the plasma after oral GlcNAc administration were significantly higher than levels before oral administration of GlcNAc. In particular, the levels of ectoine in the plasma were significantly higher after oral administration of GlcNAc than before oral administration (*p* < 0.001) in comparison to other metabolites. Considering the statistical results and the efficacy of the identified compounds, it was considered that ectoine levels might correspond to the efficacy of GlcNAc.

Ectoine is a cyclic amino acid derived from halophilic bacteria [13]. An *in vitro* study reported that ectoine is not cytotoxic, even at concentrations as high as 100 mM [14]. Another *in vitro* study reported that ectoine protects human skin epithelial cells against UVA radiation [15]. The photoprotective effects of ectoine are based upon its capacity to interfere with signaling pathways that are initiated at the cell membrane level [16].

The cumulative effects of external factors such as radiation, wind, humidity, and extreme temperatures lead to aging of the skin [17]. In a previous *in vivo* study, ectoine was shown to protect and stabilize the membranes of pre-treated cells against the damaging effects of surfactants. It also protects human skin from stress factors that would normally lead to skin dehydration [18,19]. Ectoines protect skin from the effects of UVA-induced cell damage in a number of different ways. One way in which UVA exposure causes skin damage is by ceramide formation through a singlet oxygen-mediated mechanism. The exposure of primary human keratinocytes to UVA increases ceramide levels. Consequently, an intracellular signaling cascade is activated, leading to expression of the proinflammatory intercellular adhesion molecule-1. These negative effects are blocked by ectoine through its singlet oxygen-quenching properties [15,16]. An increase in ectoine concentration induced by oral administration of GlcNAc may thus protect skin from the effects of UVA-induced cell damage.

Ectoine is also an osmoprotectant, or compatible solute, which is amphiphilic in nature and capable of wetting hydrophobic proteins. Compatible solutes may reverse osmotic inhibition because they increase the total water content and, hence increase the cytoplasmic volume of cells [20]. A recent report indicated that ectoine is useful for prevention of atopic skin dehydration, recovery of skin viability, and prevention of skin aging after topical application [19]. Therefore, increased ectoine concentrations induced by oral administration of GlcNAc may help to maintain skin moisture.

**Table 1:** Relative area before and after oral administration of GlcNAc.

Metabolites	*m*/*z*	MT	Before		After		Ratio	*p*-Value
			Mean	SD	Mean	SD		
Ectoine	143.082	9.33	1.7 × 10^−4^	6.1 × 10^−5^	9.7 × 10^−4^	5.1 × 10^−5^	5.7	7.6E-05 ***
5-Oxo-2-tetrahydrofurancarboxylic acid	129.019	10.26	2.9 × 10^−4^	9.5 × 10^−5^	1.5 × 10^−3^	1.3 × 10^−4^	5.3	0.002 **
Stachydrine	144.101	11.35	8.1 × 10^−4^	2.7 × 10^−4^	4.0 × 10^−3^	8.7 × 10^−4^	4.9	0.017 *
Trigonelline	138.055	10.33	6.8 × 10^−4^	5.0 × 10^−4^	3.0 × 10^−3^	9.7 × 10^−4^	4.4	0.035 *
1-Methyl-4-imidazoleacetic acid	141.066	8.17	1.2 × 10^−4^	4.5 × 10^−6^	5.1 × 10^−4^	5.5 × 10^−5^	4.3	0.006 **
Pimelic acid	159.066	13.79	1.6 × 10^−4^	1.1 × 10^−5^	6.6 × 10^−4^	1.9 × 10^−4^	4.1	0.044 *
XA0013	172.991	10.86	7.1 × 10^−4^	3.6 × 10^−4^	2.5 × 10^−3^	6.0 × 10^−4^	3.5	0.019 *
2-Quinolinecarboxylic acid	172.040	8.67	1.1 × 10^−4^	3.9 × 10^−5^	3.4 × 10^−4^	1.1 × 10^−4^	3.1	0.056

Asterisks indicate significant differences in the concentration of metabolites before and after oral administration of GlcNAc. *m*/*z*: mass to charge ratio. MT: migration time. All the *p* values were determined by Welch’s *t* test. *, *p* < 0.05; **, *p* < 0.01; ***, *p* < 0.001.

To our knowledge, this is the first report of increased ectoine concentrations in plasma. The reason why ectoine may be synthesized in mammals and the mechanism by which GlcNAc administration leads to an increase in ectoine plasma concentration remain unclear; future studies are required to clarify these aspects.

## 3. Methods

### 3.1. Materials

*N*-acetyl-d-glucosamine (GlcNAc) was supplied by Koyo Chemical Co., Ltd., (Tokyo, Japan).

### 3.2. Animals

Three healthy beagle dogs were used in this study. Their mean age was 4.7 years (range 4–6 years), and their mean body weight was 10 kg (range 8.8–11.7 kg). All experimental procedures performed on animals were approved by the Animal Research Committee of Tottori University.

### 3.3. Administration and Blood Sampling

A 25 mg/kg dose of GlcNAc was mixed with normal dog food (dog meal, Cainz Home Co., Ltd., Gunma, Japan). Food mixed with GlcNAc was fed at the same time every day for 35 days. The blood of each dog was collected before and 36 days after administration of GlcNAc. Blood was collected from the jugular vein using heparin as an anti-coagulant. The blood was centrifuged at 734× *g* for 10 min, and the plasma was then separated promptly and frozen at −80 °C until further analysis.

### 3.4. Metabolome Analysis

Metabolome measurements were carried out by Human Metabolome Technology Inc. Capillary electrophoresis time-of-flight mass spectrometry (CE-TOFMS) was conducted using an Agilent CE Capillary Electrophoresis System equipped with an Agilent 6210 time of flight mass spectrometer, Agilent 1100 isocratic HPLC pump, Agilent G1603A CE-MS adapter kit, and Agilent G1607A CE-ESI-MS sprayer kit (Agilent Technologies, Waldbronn, Germany). The system was controlled by Agilent G2201AA ChemStation software version B.03.01 for CE (Agilent Technologies, Waldbronn, Germany).

Cationic metabolites were analyzed with a fused silica capillary (50 μm i.d. × 80 cm total length), with a commercial cation electrophoresis buffer (Solution ID: H3301-1001, Human Metabolome Technologies) as the electrolyte. The sample was injected at a pressure of 50 mbar for 10 s (approximately 10 nL). The applied voltage was set at 27 kV. Electrospray ionization-mass spectrometry (ESI-MS) was conducted in the positive ion mode, and the capillary voltage was set at 4000 V. The spectrometer scanned from *m*/*z* 50 to 1000. Other conditions for cation analysis were performed as previously reported [21,22].

Anionic metabolites were analyzed with a fused silica capillary (50 μm i.d. × 80 cm total length), with commercial anionic electrophoresis buffer (Solution ID: H3302-1021, Human Metabolome Technologies) as the electrolyte. The sample was injected at a pressure of 50 mbar for 25 s (approximately 25 nL). The applied voltage was set at 30 kV. ESI-MS was conducted in the negative ion mode, and the capillary voltage was set at 3500 V. The spectrometer scanned from *m*/*z* 50 to 1000. Other conditions for anion analysis were performed as previously reported [22,23,24].

The raw data obtained by CE-TOFMS were processed with MasterHands [25]. Signal peaks corresponding to isotopomers, adduct ions, and other product ions of known metabolites were excluded. All signal peaks potentially corresponding to authentic compounds were extracted, and their migration times (MT) were normalized using internal standards. Thereafter, the peak alignment was performed according to the *m*/*z* values, and was normalized to the MT values. Finally, peak areas were normalized against those of the internal standards, MetSul and CSA, for cations and anions, respectively. The resultant relative area values were further normalized by sample amount. Annotation tables were produced from CE-ESI-TOFMS measurement of standard compounds, and they were aligned with the datasets according to similar *m*/*z* values and normalized MT values. Each of the metabolites were identified by referring to data which was acquired by measuring standard samples.

The metabolic pathway map was provided using public-domain software: Visualization and Analysis of Networks containing Experimental Data (VANTED, Germany) [26,27]. PCA was performed using SampleStat ver.3.14 (Human Metabolome Technologies).

### 3.5. Statistical Analysis

All values are presented as the mean ± standard deviation (SD). Welch’s *t* test was used to compare each relative area of metabolites in the plasma before and after oral administration of GlcNAc. The results were considered significant at *p* < 0.05.

## 4. Conclusions

In this study, oral administration of GlcNAc increased ectoine levels in the plasma. Increased ectoine concentration may be important for protecting the skin from the effects of UVA-induced cell damage and for maintaining skin moisture. Oral administration of GlcNAc may have restorative and protective effects that may be used in cosmetics and beauty products.

## References

[1] Chen J.-K., Shen C.-R., Liu C.-L. (2010). *N*-Acetylglucosamine: Production and Applications. Mar. Drugs.

[2] Levin R.M., Krieger N.N., Winzler R.J. (1961). Glucosamine and Acetylglucosamine Tolerance in Man. J. Lab. Clin. Med..

[3] Liu Y., Li Z., Liu G., Jia J., Li S., Yu C. (2008). Liquid Chromatography-Tandem Mass Spectrometry Method for Determination of *N*-Acetylglucosamine Concentration in Human Plasma. J. Chromatogr. B: Anal. Technol. Biomed. Life Sci..

[4] Azuma K., Osaki T., Tsuka T., Imagawa T., Okamoto Y., Takamori Y., Minami S. (2011). Effects of oral glucose hydrochloride administration on plasma free amino acid concentrations in dogs. Mar. Drugs.

[5] Tamai Y., Miyatake K., Okamoto Y., Takamori Y., Sakamoto K., Minami S. (2003). Enhanced Healing of Cartilaginous Injuries by *N*-Acetyl-d-glucosamine and Glucuronic Acid. Carbohydr. Polym..

[6] Azuma K., Osaki T., Wakuda T., Tsuka T., Imagawa T., Okamoto Y., Minami S. (2012). Suppressive effects of *N*-Acetyl-d-Glucosamine in rheumatoid arthritis mouse models. Inflamation.

[7] Salvatore S., Heuschkel R., Tomlin S., Davies S.E., Edwards S., Walker-Smith J.A., French I., Murch S.H. (2000). A pilot study of N-acetyl glucosamine, a nutritional substrate for glycosaminoglycan synthesis, in paediatric chronic inflammatory bowel disease. Aliment. Pharmacol. Ther..

[8] Chen R.H., Hsu C.N., Chung M.Y., Tsai W.L., Liu C.H. (2008). Effect of Different Concentrations of Collagen, Ceramides, *N*-acetyl glucosamine, or Their Mixture on Enhancing the Proliferation of Keratinocytes, Fibroblasts and the Secretion of Collagen and/or the Expression of mRNA of Type I Collagen. J. Food Drug Anal..

[9] Konopka J.B. (2012). *N*-acetylglucosamine functions in cell signaling. Scientifica.

[10] Dessì A., Marincola F.C., Masili A., Gazzolo D., Fanos V. (2014). Clinical Metabolomics and Nutrition: The new frontier in neonatology and pediatrics. BioMed. Res..

[11] Osaki T., Azuma K., Kurozumi S., Takamori Y., Tsuka T., Imagawa T., Okamoto Y., Minami S. (2012). Metabolomic Analysis of Blood Plasma after Oral Administration of d-Glucosamine Hydrochloride to Dogs. Mar. Drugs.

[12] Ringnér M. (2008). What is principal component analysis?. Nat. Biotechnol..

[13] Galinski E.A., Pfeiffer H.-P., Tru ¨per H.G. (1985). 1,4,5,6-Tetrahydro-2 methyl-4-pyrimidinecarboxylic acid: A novel cyclic amino acid from halophilic bacteria of the genus Ectothiorhodospira. Eur. J. Biochem..

[14] Kanapathipillai M., Lentzen G., Sierks M., Park C.B. (2005). Ectoine and hydroxyectoine inhibit aggregation and neurotoxicity of Alzheimer’s b-amyloid. FEBS Lett..

[15] Buenger J., Driller H. (2004). Ectoin: An effective natural substance to prevent UVA-induced premature photoaging. Skin Pharmacol. Physiol..

[16] Grether-Beck S., Timmer A., Felsner I., Brenden H., Brammertz D., Krutmann J. (2005). Ultraviolet A-induced signaling involves a ceramide mediated autocrine loop leading to ceramide *de novo* synthesis. J. Investig. Dermatol..

[17] Rabe J.H., Mamelak A.J., McElgunn P.J.S., Morison W.L., Sauder D.N. (2006). Photoaging: mechanisms and repair. J. Am. Acad. Dermatol..

[18] Bunger J. (1999). Ectoine added protection and care for the skin. Eur. Cosmet..

[19] Graf R., Anzali S., Buenger J., Pfluecker F., Driller H. (2008). The multifunctional role of ectoine as a natural cell protectant. Clin. Dermatol..

[20] Pastor J.M., Salvador M., Argandoña M., Bernal V., Reina-Bueno M., Csonka L.N., Iborra J.L., Vargas C., Nieto J.J., Cánovas M. (2010). Ectoines in cell stress protection: Uses and biotechnological production. Biotechnol. Adv..

[21] Soga T., Heiger D.N. (2000). Amino acid analysis by capillary electrophoresis electrospray ionization mass spectrometry. Anal. Chem..

[22] Soga T., Ohashi Y., Ueno Y., Naraoka H., Tomita M., Nishioka T. (2003). Quantitative metabolome analysis using capillary electrophoresis mass spectrometry. J. Proteome Res..

[23] Soga T., Ueno Y., Naraoka H., Ohashi Y., Tomita M., Nishioka T. (2002). Simultaneous determination of anionic intermediates for *Bacillus subtilis* metabolic pathways by capillary electrophoresis electrospray ionization mass spectrometry. Anal. Chem..

[24] Soga T., Ishikawa T., Igarashi S., Sugawara K., Kakazu Y., Tomita M. (2007). Analysis of nucleotides by pressure-assisted capillary electrophoresis-mass spectrometry using silanol mask technique. J. Chromatogr. A.

[25] Sugimoto M., Hirayama A., Ihiskawa T., Baran R., Robert M., Uehara K., Soga T., Tomita M. (2010). Differential Metabolomics Software for Capillary Electrophoresis-Mass Spectrometry Data Analysis. Metabolomics.

[26] Junker B.H., Klukas C., Schreiber F. (2006). VANTED: A system for advanced data analysis and visualization in the context of biological networks. BMC Bioinform..

[27] Klukas C., Schreiber F. (2010). Integration of -omics data and networks for biomedical research with VANTED. J. Integr. Bioinform..

